# Developing and testing guidance to support researchers engaging patient partners in health-related research

**DOI:** 10.1186/s40900-022-00378-2

**Published:** 2022-08-26

**Authors:** Jeanette Finderup, Louise Engelbrecht Buur, Sarah Cecilie Tscherning, Annesofie Lunde Jensen, Anne Wilhøft Kristensen, Anne Poder Petersen, Bente Skovsby Toft, Gitte Susanne Rasmussen, Pernille Skovlund, Tina Wang Vedelø, Lotte Ørneborg Rodkjær

**Affiliations:** 1 Department of Renal Medicine, Aarhus University Hospital, Palle Juul-Jensens Boulevard 99 Aarhus N, 8200 Aarhus, Denmark; 2 Department of Clinical Medicine, Aarhus University, Aarhus, Denmark; 3 Research Centre for Patient Involvement, Aarhus University and Central Denmark Region, Aarhus, Denmark; 4 Department of Public Health, Aarhus University, Aarhus, Denmark; 5 Steno Diabetes Centre Aarhus, Aarhus University Hospital, Aarhus, Denmark; 6 Danish Centre for Particle Therapy, Aarhus University Hospital, Aarhus, Denmark; 7 Department of Respiratory Diseases and Allergy, Aarhus University Hospital, Aarhus, Denmark; 8 Department of Dermatology and Venerology, Aarhus University Hospital, Aarhus, Denmark; 9 Department of Oncology, Aarhus University Hospital, Aarhus, Denmark; 10 Department of Neurosurgery, Aarhus University Hospital, Aarhus, Denmark; 11 Department of Infectious Diseases, Aarhus University Hospital, Aarhus, Denmark

**Keywords:** Patient and public involvement, Patient partner, Patient engagement, Framework, Health-related research

## Abstract

**Background:**

Although interest in Patient and Public Involvement in health-related research is growing, there seems to be a lack of guidance supporting researchers in deciding on methods and levels for Patient and Public Involvement in health-related research throughout the research process. Furthermore, the numerous definitions, methods, and frameworks make it challenging for researchers new to this field to decide on the most appropriate approach for their project.

**Methods:**

This study aimed to develop and test guidance for researchers deciding on approaches, levels, and methods for engaging patient partners in health-related research. A group of 11 researchers in Patient and Public Involvement in health-related research participated in six workshops to develop the guidance. The feasibility and acceptability of the guidance were tested in a survey of 14 researchers using the System Usability Scale plus two elaborative questions. The guidance was also tested by five PhD students engaging patient partners in their projects.

**Results:**

The guidance developed consisted of two resources: Resource I outlined five international approaches to Patient and Public Involvement in health-related research, and Resource II described the different levels and methods for engaging patient partners in research. The System Usability Scale score (at the 50th percentile) was 80, indicating excellent usability. Qualitative data showed that the two resources supported reflections regarding different approaches, levels, and methods.

**Conclusion:**

The researchers found the guidance to be supportive of their reflective thinking about engaging patient partners in their research. The testing provided knowledge about when and how to use the guidance but also raised questions about the usefulness of the guidance in communications with patients.

**Supplementary Information:**

The online version contains supplementary material available at 10.1186/s40900-022-00378-2.

## Plain English summary

More and more patients are taking part in research as patient partners. However, researchers have little guidance on how to bring patient partners into research studies and at what levels. There are many ways to do this. It can be hard to choose the way most appropriate for a specific project, especially for newer researchers. We (11 researchers at different career levels) developed guidance through six workshops to help new researchers choosing the way to engage patient partners most appropriate for their project. The guidance resulted in two different resources. Resource I gives five international approaches for bringing patient partners into research. Resource II describes methods for engaging patient partners at different levels. Fourteen researchers and five PhD students tested the guidance and scored how usable it is. It had excellent usability. The two resources did help researchers to decide on the best ways to engage patient partners in research. We now need to test how the guidance can be used to talk to patients about taking part in specific research projects.

**Supplementary Information:**

The online version contains supplementary material available at 10.1186/s40900-022-00378-2.

## Background

Patient and Public Involvement in health-related research (PPI) is a topic of increasing interest internationally. In general, PPI refers to the engagement of patients, relatives, the public, and other stakeholders in the research process [1]. The rationale for engaging patient partners as collaborative partners in research on an equal and structural basis may be summarized as follows: (1) Patients have the democratic right to be engaged in research on their health condition, and researchers have a moral imperative to ensure their engagement; (2) Bringing a lifeworld perspective into the research design and delivery may improve the research quality by increasing relevance and improving recruitment and retention rates; and (3) Co-constructed knowledge by patients and researchers enhances accountability and transparency [1]. Many international organizations have put patient partner engagement on their agenda, especially in the UK, the USA, Canada and Australia, where PPI is well established [2, 3]. There is well-known peer-reviewed literature on PPI internationally [3, 4], but in Europe most publications are from the UK [1], and a lack of publications from continental Europe has been identified [5]. The different cultural approaches to PPI across European countries may partly be the reason for this and the lack of leading organizations promoting PPI in some parts of the continent. From a Nordic perspective, researchers indicated a lack of knowledge about methods for PPI and the impact of PPI in research [6]. The increasing number of internationals PPI publications often report descriptions of principles and best-practice activities [7] and frameworks [1]. Hence, in the review by Greenhalgh et al. [1], 65 frameworks from ten different countries were identified for research supporting, evaluating, and reporting PPI. The different terminologies used may be confusing as there is little consensus in the literature on the use of terms such as "involvement", "engagement", and "patient-oriented research", raising the question of which definition should be considered most appropriate. Similarly, there is no agreement on whether "patient", "partner", or "patient partner" is the most suitable term [8].

Furthermore, frameworks developed to guide PPI in specific contexts were seldom transferable to other contexts unless they were oriented to and used in a specific clinical field [1]. Researchers argue that it is challenging to navigate the complex field of PPI. They find it difficult to formulate concrete and uniform answers to questions about whom to engage and when, and how to engage patient partners in health-related research [4, 9]. Others highlight that much of the existing guidance is generic and that researchers are not always clear about how to apply the general advice to the specifics of their work [10]. There seems to be a lack of literature available on applying the various concepts of PPI in practice when collaborating in a structural manner with patient partners [11, 12]. In addition, researchers have pointed out several dilemmas and challenges related to the role and responsibility of initiating and facilitating PPI in health-related research projects [13–15]. Researchers may be hesitant regarding the approach to choose and the desired level of patient partner engagement in their projects or in which phases of the research process it would become a meaningful task to engage patient partners [16] and a need for training and guidance have been voiced [17]. According to de Wit et al. the basic research curriculum of PhD candidates seldom contains building competence on PPI [16]. In order to increase the quality, credibility and uptake of PPI research, initiatives have been taken to train and guide researchers e.g., the FIRST model [18], the course “Foundations in Patient-Oriented Research” [19], and the “Preparing researchers for user involvement” programme [16].

Furthermore it seems that PPI can be conducted at different levels. According to Arnstein’s ladder of citizen participation [20] and the Pathways to Participation model [21–23], a "higher" level of PPI can be regarded as more valuable than a "lower" level. In addition, a horizontal level of involvement has been described in Health Canada's Public Involvement Continuum [24]. Here, no level is valued over another, but it is rather a matter of choosing the most suitable level and methods depending on the aim of PPI in the individual project. An "Involvement Matrix" has been published, focusing on clarifying the roles of patient partners and aligning mutual expectations for a project [25]. However, there still seems to be a need to establish a more common understanding of approaches, levels, and methods for PPI and, moreover, how this could be further conceptualized [26]. It has been suggested that researchers need to select and adapt existing frameworks and guidelines to meet the needs of their research and context [1]. However, to date, limited efforts have been made to summarize the key approaches and methods from these conceptual frameworks and provide some best-practice recommendations. Therefore, there is a need for guidance to support researchers new to PPI. An online resource that signposts researchers to the most relevant guidance and key resources may be of great value, considering that this kind of guidance is generally of the greatest value to early career researchers [9]. Well aware that researchers also need training and practical guidance to become skilled researchers practising meaningful PPI due to mutual learning processes with the patient partners [18].

In Denmark, PPI is a new discipline compared to the UK, the USA, Canada, and Australia. In 2016, the extent of PPI across the country was mapped, and the findings indicated that, since 2014, PPI has become integral to health-related research [27]. As several Danish patient associations and health-related research funds have begun to require a statement on PPI in their calls for research proposals, there is an increasing demand for guidance to support researchers. In Denmark, no national approach to PPI exists, forcing Danish researchers to look to international approaches for guidance. As a newly established network of researchers engaging patient partners in health-related research, we have experienced this need for an overview of the various essential concepts and the literature exploring these concepts.

This paper contributes to the existing literature by evaluating the usefulness of developing guidance to support researchers deciding on which PPI approaches to choose for their research, and the creation of an overview of some methods for engaging patient partners at different levels in health-related research. To our knowledge, this guidance is the first Nordic attempt to support researchers who either plan to engage or are engaging patient partners in their research projects.

## Methods

This study aimed to develop and test guidance for researchers deciding on approaches, levels, and methods for engaging patient partners in health-related research. The study was supported by the Research Centre for Patient Involvement (ResCenPI), which was established in the Central Denmark Region in 2019 [28]. ResCenPI investigates interventions helping patients, relatives, and health professionals to be involved effectively in health care of relevance to people’s daily lives. One of ResCenPI’s main research areas is exploring methods for the meaningful involvement of patients and other relevant stakeholders throughout the research process.

### Development of PPI guidance

A cross-disciplinary collaborative network was established within ResCenPI. The network consisted of 11 healthcare researchers (authors of the present paper). The researcher representing different clinical fields; oncology, nephrology, endocrinology, dermatology and infectious diseases and they share a special interest in PPI. The members had various levels of research experience, ranging from senior researchers to PhD students. Moreover, the experience and expertise within PPI research varied in the network. The senior researchers have all published PPI related research. All the PhD students are principal investigators in ongoing studies engaging patient partners in the research processes. No patient representatives were included in the development of the PPI guidance, as the collaborative network started as a network for researchers. Through these discussions, the need for a PPI guidance supporting researchers appeared. In the light of this, researchers and PhD students were considered as the end-users of the PPI guidance and were involved in the present development and evaluation.

The network worked collaboratively via a series of workshops fall 2020 and spring 2021 to develop PPI guidance for researchers engaging patient partners in health-related research. The workshops were either physical or online meetings and lasted for approximately two hours each. To ensure the work progressed, each member was responsible for preparing and presenting a delegated assignment. The content of these presentations established a starting point for further discussions and decisions, and for seeking consensus in the workshops. At the end of each workshop, the members agreed the delegated assignments for the next workshop. The preparation time for each member was approximately four hours before each workshop. An overview of the tasks and content of the workshops is presented in Table 1.

**Table 1:** Overview of tasks and content of PPI workshops

Workshop	Task	Content
Development of PPI guidance		
1	PPI approaches – facts	Identification and selection of relevant PPI approaches [4]
2	PPI approaches – what and why	Discussion of PPI approaches: definition, mission, and vision within the selected PPI approaches
3	PPI methods – how to	Discussion and agreement on appropriate methods for PPI inspired by Health Canada’s Public Involvement Continuum [17]
4	Development and finalization of PPI guidance	Decisions on layout, design, and format of the PPI guidance
Testing of PPI guidance by health-related researchers		
5	Usability and end-user experiences of PPI guidance	Discussion and agreement on methods for testing usability and investigating end-user experiences
6	Analysis and results	Discussion of results from testing of the PPI guidance

### Testing of the PPI guidance

The usability and end-user experiences of the PPI guidance were tested during fall 2021 using various methods. Firstly, a survey was conducted among members of ResCenPI. Secondly, PhD students provided their experiences of using the PPI guidance by replying to open-ended questions in writing.

### Usability test

**Fig. 1:**
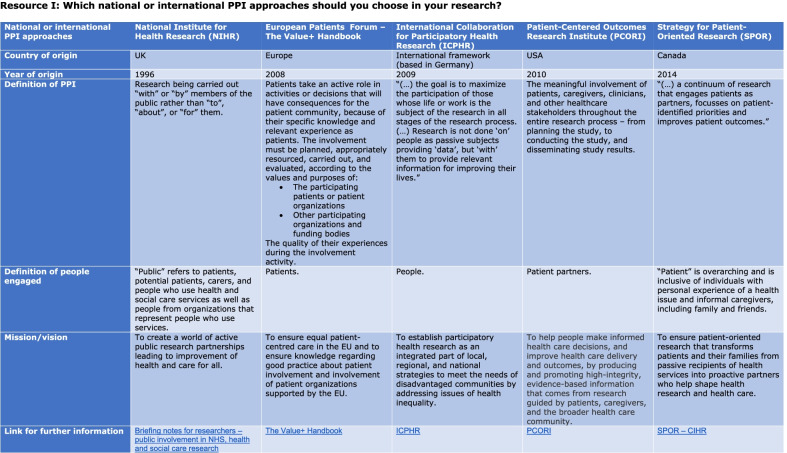
Resource I Which national or international PPI approaches should you choose in your research?

**Fig. 2:**
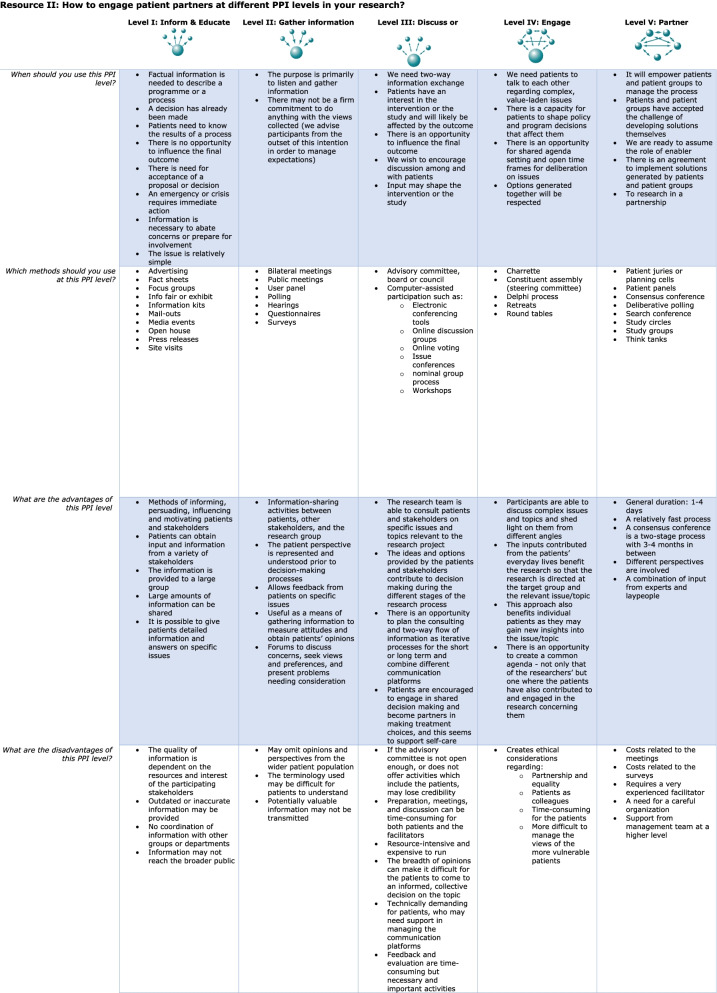
Resource II How to engage patient partners at different PPI levels in your research?

Members of ResCenPI were invited by email to test the PPI guidance in October 2021. The participants had one month to test and evaluate the PPI guidance. Within this time span, one reminder was sent. In the email, the participants received the PPI guidance (Figs. 1, 2) and a questionnaire. The questionnaire consisted of the System Usability Scale (SUS) [29, 30] and two additional open-ended questions (Additional file 1). The usability of the PPI guidance was tested with SUS. This scale is validated to measure the usability of interventions and is also reliable with small sample sizes. It consists of ten items with five response options: from strongly agree to strongly disagree. The participants completed the SUS immediately after testing the PPI guidance. For the analysis of the SUS, each item was converted to a number, summarized, and multiplied by 2.5 to give a total score between 0 and 100. Data were presented in percentile rankings. Prior studies defined a SUS score of 68 as average, and a score above 80 indicated excellent usability [31].

### End-user experiences

To explore the end-user experiences, the PPI guidance was also evaluated by five PhD students who were engaging patient partners in research. The PhD students received an email containing the PPI guidance and seven elaborative questions (Additional file 2).

Data from the two additional open-ended questions answered by the members of ResCenPI and the responses from the PhD students were analysed using thematic analysis [32]. The analysis sought to establish an in-depth understanding of whether the PPI guidance supported researchers in engaging patient partners in health-related research and, if so, how. It also explored the researchers’ considerations when engaging patient partners in health-related research. Suggestions made in relation to improving the guidance were also analysed.

## Results

### Development of PPI guidance

The starting point for developing the guidance was the PPI initiatives of NIHR (National Institute for Health and Care Research) [10], PCORI (Patient-Centered Outcomes Research Institute) [33], SPOR (Strategy for Patient-Oriented Research) [34], ICPHR (International Collaboration for Participatory Health Research) [35], and Value + (Promoting Patients’ Involvement in EU Supported Health-Related Projects) [36], as described by Beresford and Russo [2]. Health Canada’s Public Involvement Continuum [24] was chosen because it describes different roles and interactions which characterise different levels, and links to specific methods of conduct at these levels. The PPI guidance we developed comprised two resources (see Figs. 1, 2):

Resource I presents five different initiatives from the UK [37], the USA [33], Canada [34], Germany [35], and Europe [36] published between 1996 and 2014. They are generic initiatives developed to guide researchers in their work with PPI within different medical specialities and settings. The different definitions of PPI and the people engaged (as what we would term patient partners) are described, as well as each initiative’s mission/vision. Resource I aims to provide an overview of the available options so researchers can find the most suitable method for their project.

Resource II presents figures that illustrate five levels of PPI (levels 1–5) on a horizontal line. The arrows between the dots represent the direction of communication, where the dot at the bottom represents the researcher, and all other dots represent the patients (or other stakeholders). Within each level, its methods, advantages, and disadvantages are listed. Even though the levels are presented separately, they supplement and overlap each other, as different strategies are often combined. Resource II aims to help researchers reflect on what the main purpose of PPI is in their research, decide if they want to inform/educate, gather information, discuss, engage, or even establish a partnership with patients, and then decide on which methods to use.

### Testing the PPI guidance

In total, 14 participants tested the guidance and answered the two elaborative questions and the SUS. The characteristics of the 14 participants are shown in Table 2. Nearly half of the participants were early career researchers (43%). Half of the participants had worked with PPI for between one year and three years, with the remainder split between those who had worked with PPI for less than one year (29%) and for three years or more (21%).

**Table 2:** Characteristics of the participants

Questions	Number (%)
How much experience do you have within research?	
Research assistant & PhD student	6 (43)
Junior researcher & Senior researcher	8 (57)
How many years have you worked with PPI?	
Less than one year	4 (29)
Between one year and three years	7 (50)
Three years or more	3 (21)

The average SUS score (at the 50th percentile) was 80, indicating excellent usability. The group of early career researchers gave an average SUS score (at the 50th percentile) of 67.5, and the group of more experienced researchers 85. Those who were less experienced with PPI gave an average SUS score (at the 50th percentile) of 78.75, the group with 1–3 years of experience 67.5, and the most experienced 87.5.

**Fig. 3:**
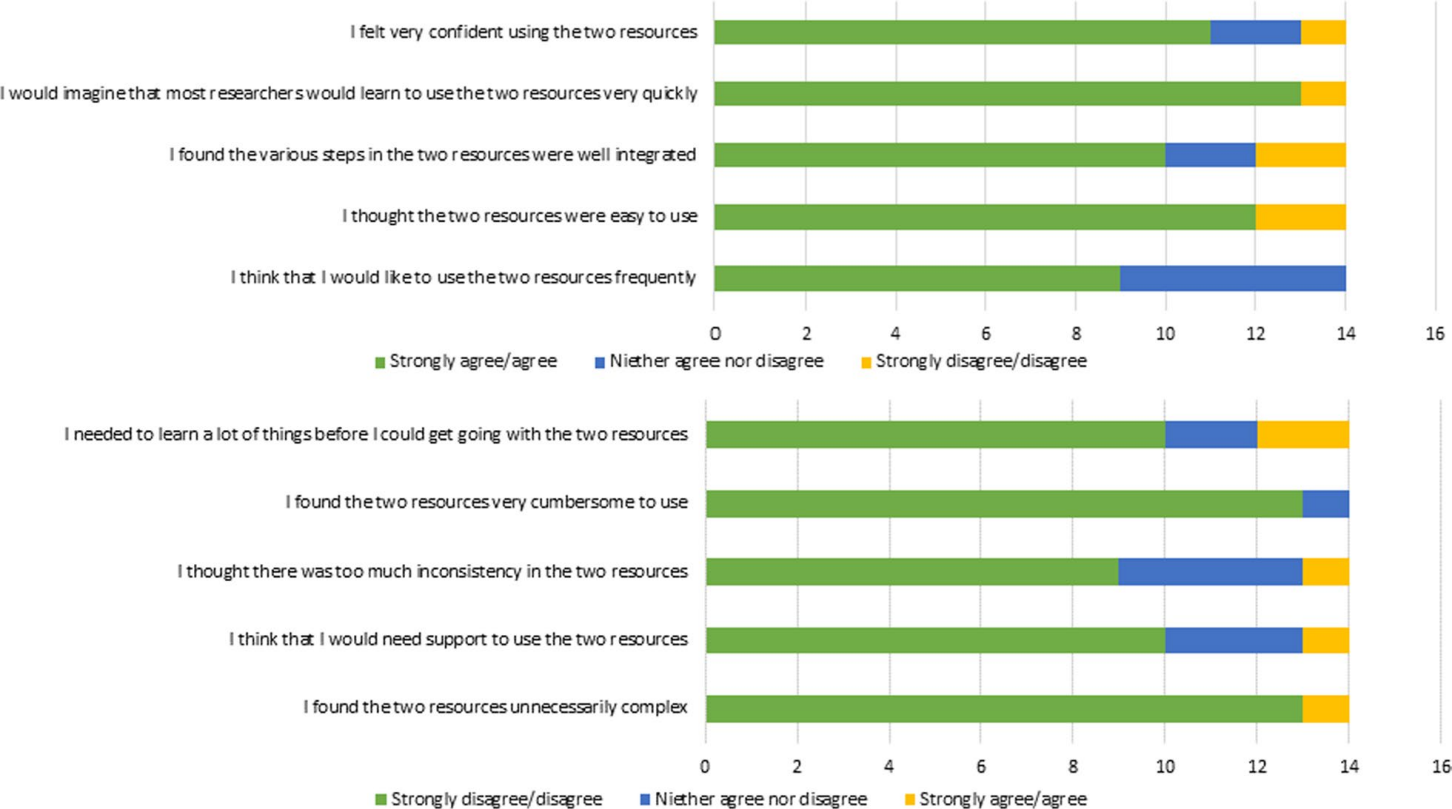
Summary of the SUS scores for each question

Figure 3 presents a summary of the SUS scores for each item, showing that the items “I would imagine that most researchers would learn to use the resources very quickly” and “I thought the two resources were easy to use” obtained the most positive scores, and one of the reversed items – “I found the two resources unnecessarily complex” – obtained the most negative score, which is also positive. The two items “I think that I would like to use the two resources frequently” and “I thought there was too much inconsistency in the two resources” obtained the lowest positive scores.

The qualitative data from the open-ended questions showed that the two resources supported reflections about the different approaches, levels, and methods. Most participants confirmed that the two resources supported them in their decision about which approach to choose. All participants found that Resource I provided them with a useful overview of the different approaches which helped them to position their research. Many commented that such an overview was hard to find elsewhere, possibly because there are so many different approaches. For example, one participant stated:

“Yes, I got an answer to the question and am now clearer about which approach supports the way I use PPI in my research.”

Being able to compare the visions/missions in Resource I was highlighted as especially valuable, as were the links to web pages giving further information.

Resource II was found to be a quick and convenient way to access an overview of the different levels of PPI. It was viewed not only as a tool which could be used at the beginning of the research process, but also later, to confirm clear definitions of which levels of PPI were involved in a project. Some researchers described it as an eye-opener that would increase their use PPI in their research or at certain stages of the research process. It was also found that Resource II could help to establish a clearer definition of the role of patient partners in the research process.

“I think the information is very useful and I would certainly use this in the future when thinking about the most effective way to integrate PPI within a research project/study. I like the use of comparison tables which summarize key points of information.”

The qualitative data primarily from the PhD students revealed how participants chose different approaches, at what level they chose to engage patient partners, and which methods they used. Here, participants stated that their choice of approach depended on the context, e.g., who they wanted to engage and how. Most participants chose to use more than one approach. If they intended to engage patient partners throughout the whole research process, then several chose to use PCORI. In contrast, when PPI was not to be used throughout the research process, then NIHR was chosen more often. SPOR was chosen by one of the participants, who commented that this approach seemed to emphasize engagement and promote patients' active engagement. The participants chose levels II, III, or IV, and three of them decided to have more than one level of PPI in their studies. One said that the level was chosen to maximize the engagement of the patient partner. There were also comments that PPI brought other perspectives into the research. Nonetheless, it seemed to be difficult for participants to give a definitive explanation for their choice of level of engagement. The participants used between one and six different methods of including PPI. All five PhD students involved an advisory board, and other methods used were workshops, participant observations, meetings, questionnaires, and interviews.

## Discussion

This study may be the first step in developing PPI guidance for health-related researchers in a Danish context – where no PPI guidance currently exists at a national level, despite some incipient initiatives [27, 38]. The guidance was developed to address the current needs of researchers for a comparison across frameworks of how to meaningfully engage patient partners in health-related research. The existence of 65 frameworks [1] and numerous different definitions of what patient partner engagement is, made it a challenging starting point. The testing of the guidance by researchers with different amounts of PPI experience showed excellent usability in terms of supporting researchers’ and PhD students’ reflections on how to decide on approaches, levels, and methods for engaging patient partners in health-related research. These results offer a good starting point for further development and future use of the PPI guidance.

The PPI guidance was developed to assist researchers in choosing appropriate approaches, methods, and levels of engaging patient partners in research. An appropriate approach may be to reflect upon definitions and mission/visions chosen, for example by using Resource I. Moreover, researchers should take in to account the methods that best acknowledge the patient partner’s relevant experiences and adapt the level of involvement (Resource II) to the patient’s qualities in terms of confidence, motivation, knowledge, skills, and willingness to be involved in order to provide an authentic patient perspective (24). It was co-created in a local context through collaborative processes by a cross-disciplinary network of 11 researchers with both a special interest and varying research experiences in PPI. The process entailed a comprehensive review of the literature and in-depth reflections and discussions prior to developing the guidance.

Resource I comprised an overview of existing approaches, which may help researchers to reflect on and choose the most appropriate approach for their research project. Resource I was based on the different approaches of five organizations: NIHR [37], PCORI [33], SPOR [34], ICPHR [35], and Value + [36], which were identified as central by Beresford and Russo [2]. These approaches were found to be relevant and represented different foci (e.g., the partnership-focused approach of NIHR [37] or the priority focus of SPOR [34]). We acknowledge that other organizations and approaches exist. However, we consider this to be an appropriate starting point for more embedded and formalized implementation of PPI, using infrastructure, organizational support, guidelines, and resources, as suggested by Biddle et al. [5]. This resource may meet the growing interest in the idea of PPI, and it may also be helpful for future applications if EU funders’ perception of PPI develops and its implementation changes from a recommendation to a requirement [5].

Resource II presented five different levels of engagement and methods of collaboration, ranging from information and education to partnership. It was developed to establish the key concepts, principles, and areas for patient partner engagement that would ideally be adopted by more stakeholders. It was based on Health Canada’s Public Involvement Continuum [24], which was found to foster understanding due to its horizontal orientation reflecting a continuum rather than stepwise involvement. However, there is discussion in the literature of whether the actions at levels one and two can truly be considered involvement or if they are merely tokenism [20]. At level one, patients are informed of the results of a process, but they have no opportunity to influence it. At level two, patients give information to the researchers, but the researchers make no commitment to use it. The main problem may be that the actions at levels one and two represent one-way communication and do not offer the possibility of negotiation and real influence [20]. An additional problem may be that Health Canada’s Public Involvement Continuum [24] was originally developed for the involvement of citizens in government decision making on health issues. Therefore, the question of whether levels one and two – where patients are not offered a real opportunity to influence the research – are applicable to PPI is still open to discussion. On the other hand, in some situations, levels one and two may be the only way to engage patient partners. In one study with frail older patients, the researchers intended to engage the patients at higher levels, but due to the patients’ frailty, they found that it was not possible [39]. This highlights the need for flexible and adjustable methods of engaging patient partners in a transparent way. In this study, we have focused on the development of a guidance for health-related researchers based on formal learning, therefore the resources will only contribute to the formal learning of the researchers, though we acknowledge that experiential and social learning is also needed [40].

Staley et al. [9] emphasize the importance of adapting patient partner engagement in research to the specific context. Based on their multinational (Canada, Australia, the UK, and USA) study, Concannon et al. [41] offer practical guidance on designing and implementing an engagement plan using the generic model “plan-do-study-act”, which can be adapted to the local context. They present a matrix to summarize engagement activities and a list of reflective questions to assist in selecting appropriate roles and modes of engagement [41]. However, they do not explicitly discuss this approach in relation to other frameworks and their divergent definitions of PPI. National guidance on “Patient and Public Involvement and Engagement” has been developed in Austria by a research group using a multi-stakeholder approach of co-creation with similar processes to this study. However, they chose to use the NIHR definition [42] and Arnstein model [20] of different levels of engagement in their guidance [43]. The review by Greenhalgh et al. [1] highlighted that there are numerous published frameworks to support PPI that have been developed in different contexts. However, no single framework suits every case, and each framework is primarily used by those who developed it. They conclude that no “one-size-fits-all” approach is likely to succeed and recommend selecting and adapting existing frameworks to meet one’s own needs and context. This supports the idea of developing PPI guidance in a local context. Nonetheless, there may still be a need to consider the perspectives of other researchers in Denmark and test this study’s PPI guidance in other health-related research settings.

Previously, an involvement matrix had been developed as a tool to support PPI [25], and we suggest that future research should be directed towards developing a generic tool based on Resource II that can be of practical use in designing and evaluating the level of PPI in Danish research studies regardless of the specific framework and approaches used. A further goal would be to develop a systematic approach to monitoring the extent and impact of PPI. Just as important it is to recognize that researchers continue to struggle with how to operationalise research partnerships with patients, both practically and effectively [44]. For example, in the UK it has been proposed by INVOLVE, that training should be provided for both patient partners and researchers in terms of activities that aims develop knowledge, skills and experience that prepare them for PPI [45]. In the Netherlands, the FIRST model has been suggested to be used as a framework for establishing a structural partnership with the patient partners [18]. This may be an inspiration for bringing PPI forward in Denmark in collaboration with the patient partners.

### Limitations

One limitation of this study is that the PPI guidance developed is preliminary and may benefit from other stakeholders’ perspectives and from being tested in different settings and by researchers in different disciplines. Another limitation may be that we considered the researchers and PhD students to be the end-users, which is why we did not include patients in the development process and testing. However, in future development of the guidance, it will be important to include patients as they are key in terms of communication about ways to support patient partnerships and valuing the patient partner role [46]. Another limitation is that the PPI guidance was not qualitative evaluated with in-depth interviews. This could have nuanced the perspectives of the end-users and their suggestions for improvement. Furthermore, it could have been beneficial to evaluate Resource 1 and Resource 2 separately, as Resource 2 is a more cognitively demanding tool to guide researchers in PPI than the more straight forward and didactic Resource 1.

## Conclusion

This study has developed two resources to guide researchers in engaging patient partners in health-related research. The study provides insights into the collaborative processes of developing context-specific PPI guidance based on existing approaches and guidelines. Testing of the PPI guidance among researchers showed that they found it to be useful in facilitating reflective thinking around engaging patient partners in health-related research. The study provided knowledge about when and how to use the guidance, but also raised questions about whether the PPI guidance may be useful in communications with patients.

## Supplementary Information

**Additional file 1.** The questionnaire sent out consisting of the System Usability Scale and two additional open-ended questions.

**Additional file 2.** The seven elaborative questions sent out for the PhD students.

## Data Availability

The data that support the findings of this study are available from the first author, but restrictions apply to the availability of these data, which were used under licence for the current study, and so are not publicly available. Data are however available from the authors upon reasonable request and with the permission of the first author.

## Abbreviations

PPI: Patient and public involvement in health-related research
SUS: System usability scale
PCORI: Patient-centered outcomes research institute
NIHR: National institute for health and care research
SPOR: Strategy for patient-oriented research
VALUE +: Promoting patients’ involvement in EU supported health-related projects
ICPHR: International collaboration for participatory health research
ResCenPI: Research centre for patient involvement
