# Supplementary material for: Developing and testing guidance to support researchers engaging patient partners in health-related research
Source: Res Involv Engagem. 2022 Aug 26;8:43. doi: 10.1186/s40900-022-00378-2 (PMC9413931; doi:10.1186/s40900-022-00378-2)
Supplement: Supplementary file 1 — Additional file 1. The questionnaire sent out consisting of the System Usability Scale and two additional open-ended questions. [file 40900_2022_378_MOESM1_ESM.docx]

**Additional material I**

Questionnaire consisting of the System Usability Scale and two additional open-ended questions

**Patient and public involvement in health-related research (PPI) resources**

Dear Researcher,

In the Research Centre for Patient Involvement (ResCenPI), we have developed two resources to inform investigators about patient and public involvement in health-related research (PPI). 

1. A one-page resource outlines five national and international PPI approaches to consider.
2. A two-page resource describes the different levels and methods of engaging patient partners in your research project.

We would value your feedback on these resources. Your anonymized answers will be used to modify the resources, and inform a publication on how to support investigators to engage patient partners in research projects.

The target groups for the resources are researchers who plan to engage patient partners in their research projects or researchers who already engage patient partners in their research projects.

To foster meaningful PPI, the purpose of the two resources is to support researchers in deciding on approaches and methods for engaging patient partners in health-related research. Their aim is to answer the following two questions:

1) Which national or international PPI approaches should you choose for your research?
2) How should you engage patient partners at different PPI levels in your research?

**A: Testing the two resources**
Please use the two resources to answer these two questions.

**B: Evaluating the guidance**
Afterwards, we would like you to answer this questionnaire, which consist of 14 questions.

The deadline for participating in the test and evaluation is 15th of November 2021.

Best regards
The PPI network in ResCenPI


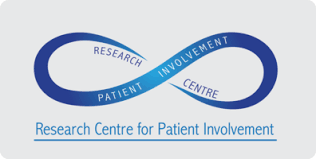


| **How much experience do you have within research?** |
| --- |
| - Research assistant - PhD student - Junior researcher (PhD, post-doc fellow, assistant professor) - Senior researcher (associate professor, professor) - Other |

| **How many years have you worked with PPI?** |
| --- |
| - Less than one year - Between one year and three years - Three years or more |

| **Using Resource I, did you reach an answer to the question:**  *Which national or international PPI approaches should you choose for your research?*  **In what way did Resource I help you to answer the question? Please elaborate below.**   \|  \| \| --- \| |
| --- | --- |
| **Using Resource II, did you reach an answer to the question:**  *How should you engage patient partners at different PPI levels in your research?*  **In what way did Resource II help you to answer the question? Please elaborate below.**   \|  \| \| --- \| |

**We will now present you with ten different statements and we would like you to rate each statement from strongly agree to strongly disagree.**

**I think that I would like to use the two resources frequently.**

- Strongly agree
- Agree
- Neither agree nor disagree
- Disagree
- Strongly disagree

**I found the two resources unnecessarily complex.**

- Strongly agree
- Agree
- Neither agree nor disagree
- Disagree
- Strongly disagree

**I thought the two resources were easy to use.**

- Strongly agree
- Agree
- Neither agree nor disagree
- Disagree
- Strongly disagree

**I think that I would need support to use the two resources.**

- Strongly agree
- Agree
- Neither agree nor disagree
- Disagree
- Strongly disagree

**I found the various steps in the two resources were well integrated.**

- Strongly agree
- Agree
- Neither agree nor disagree
- Disagree
- Strongly disagree

**I thought there was too much inconsistency in the two resources.**

- Strongly agree
- Agree
- Neither agree nor disagree
- Disagree
- Strongly disagree

**I would imagine that most researchers would learn to use the two resources very quickly.**

- Strongly agree
- Agree
- Neither agree nor disagree
- Disagree
- Strongly disagree

**I found the two resources very cumbersome to use.**

- Strongly agree
- Agree
- Neither agree nor disagree
- Disagree
- Strongly disagree

**I felt very confident using the two resources.**

- Strongly agree
- Agree
- Neither agree nor disagree
- Disagree
- Strongly disagree

**I needed to learn a lot of things before I could get going with the two resources.**

- Strongly agree
- Agree
- Neither agree nor disagree
- Disagree
- Strongly disagree

**If we may contact you for elaboration on your answers, please write your name and phone number in the box.**

|  |
| --- |

**Thank you for answering the questions!

Best regards
The PPI network in ResCenPI


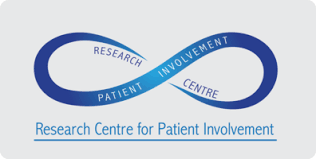
**
