# Supplementary material for: Developing and testing guidance to support researchers engaging patient partners in health-related research
Source: Res Involv Engagem. 2022 Aug 26;8:43. doi: 10.1186/s40900-022-00378-2 (PMC9413931; doi:10.1186/s40900-022-00378-2)
Supplement: Supplementary file 2 — Additional file 2. The seven elaborative questions sent out for the PhD students. [file 40900_2022_378_MOESM2_ESM.docx]

**Additional material II**

**Questionnaire on how PhD students use PPI^[[1]](#footnote-1)^ in their research**

| Template for reporting on how you use PPI in your research |
| --- |
| **Which PPI approaches did you choose, or would you choose?** |
| **Why?** |
| **Which levels of involvement did you choose, or would you choose?** |
| **Why?** |
| **Which methods for involvement did you choose, or would you choose?** |
| **Why?** |
| **Which reflections have the two resources contributed to?** |

1. Patient and Public Involvement in health-related research [↑](#footnote-ref-1)
